# Comment on: Tan WJ, Cima I, Choudhury Y, Wei X, Lim JCT, Thicke AA, Tan MH, Tan PH. A five-gene reverse transcription-PCR assay for pre-operative classification of breast fibroepithelial lesions. Breast Cancer Research 2016;18:31

**DOI:** 10.1186/s13058-016-0730-4

**Published:** 2016-07-25

**Authors:** Joseph Loane

**Affiliations:** Western General Hospital, Crewe Road, Edinburgh, UK

I read with interest the article by Tan et al. [1] describing a five-gene reverse transcription-PCR assay for pre-operative classification of breast fibroepithelial lesions (FELs).

I would like to raise three points, however: one regarding the presentation of the statistics in this paper, and two about the practical application of this assay.

Firstly, it is not clear whether the test group is representative of the prevalence of phyllodes tumours (PT) in their population. Because the rate of cases diagnosed as fibroadenoma (FA) on core biopsy with a subsequent diagnosis of PT on excision in their test group is more than four times that which they reported previously as part of a multi-centre study on a series of 4163 consecutive such cases [2], it would appear more likely that the test group is not representative. If this is the case, their calculation of the positive predictive value (PPV) and the negative predictive value (NPV) (which are dependent on disease prevalence) is in error and leaves it unclear how this assay would perform in practice.

Secondly, in relation to Tan et al.’s indeterminate FELs on core biopsy, because 21 of the 22 of these in their test group turned out to be PTs on excision, their histological classification as indeterminate FELs on core biopsy performed better than their five-gene assay at predicting PTs on excision (94.5 % vs 82 %). Again, however, this is likely to be a selected group, as the authors quote a rate of 41–63 % for these cases in their previous publication [2].

Finally, regarding the rare cases which on excision turn out to be PT following a histological diagnosis of FA on core biopsy—there were only 16 such cases in their previous study of 4163 consecutive cases—the practicality of applying this molecular assay in identifying these cases is questionable. To identify one true positive, 260 cases would need to be tested, and it is likely that testing would generate more false positive than true positive results.

While it remains an interesting avenue of investigation for these challenging lesions, the general applicability of the findings in this study appears limited by its design and by the practical difficulties inherent in identifying very rare events. It may be that, as yet, what we know of the molecular characteristics of this complex group of tumours does not add enough to be of practical value in their pre-operative classification.
