# Supplementary material for: Comment on: Tan WJ, Cima I, Choudhury Y, Wei X, Lim JCT, Thicke AA, Tan MH, Tan PH. A five-gene reverse transcription-PCR assay for pre-operative classification of breast fibroepithelial lesions. Breast Cancer Research 2016;18:31
Source: Breast Cancer Res. 2016 Jul 25;18:77. doi: 10.1186/s13058-016-0730-4 (PMC4959056; doi:10.1186/s13058-016-0730-4)
Supplement: Related texts

# Reply: Authors’ response

**Authors:** Wai Jin Tan, Min-Han Tan, Puay Hoon Tan

We appreciate the comments offered by Dr Joseph Loane on our publication [1]. To address the issues raised, it is important to describe the cohort involved in our paper and that of the multi-centre study [2]. Our assay was developed using an initial test set of 48 samples of FAs and PTs, including paired core biopsies with subsequent excisions. This was then validated on 230 core biopsies, of which 131 were diagnosed as FAs with event-free follow-up of at least 2 years, and on 99 FAs and PTs (*n* = 58 and *n* = 41, respectively) concluded by surgical excisions with corresponding pre-operative core biopsies, including 22 cases in which the core biopsy diagnosis was indeterminate. A large proportion of core biopsies with subsequent excisions serving as the reference diagnosis was selected. In contrast, the multi-centre study reviewed consecutive core biopsies diagnosed as unequivocal FAs which would not ordinarily be excised, determining the likelihood of subsequent occurrence of PT on follow-up as 0.38 %. This study concluded that a firm diagnosis of unambiguous FA on core biopsy was reliable and safe with adequate imaging and follow-up [2]. It is therefore not unexpected that the rate of subsequent diagnosis of PT among core biopsies in our validation cohort was substantially higher than in the multi-centre study, as the aim and selection criteria were different. The PPV and NPV calculated in our study were based on the validation cohort used, and we agree that an optimal PPV and NPV could be derived from application of the assay in a prospective hospital-based cohort evaluating key outcomes including recurrence. This would be best addressed by a follow-up study. Notably, PTs are more common in Asian women [3].

The indeterminate FELs diagnosed pre-operatively were histologically inconclusive; it is unclear how indeterminate calls could be compared or inferred as performing better than the assay. Discordance (4/22; 18 %) between the assay and reference diagnosis among FELs may reflect tumour heterogeneity because the assay was performed on limited core biopsy material, while the reference diagnosis was based on surgical excisions.

Distinguishing cellular FELs on core biopsy into FA vs PT remains challenging, with multiple studies attempting to use a variety of microscopic parameters, none of which are uniformly predictive [4, 5]. An adjunctive objective practical assay such as ours to aid decision-making will be helpful, especially if further validated in larger studies in a multi-centre prospective setting.

## Abbreviations

FA, fibroadenoma; FEL, fibroepithelial lesion; NPV, negative predictive value; PCR, polymerase chain reaction; PPV, positive predictive value; PT, phyllodes tumour
